# Effect of the macroalgae *Asparagopsis taxiformis* on methane production and rumen microbiome assemblage

**DOI:** 10.1186/s42523-019-0004-4

**Published:** 2019-02-12

**Authors:** Breanna Michell Roque, Charles Garrett Brooke, Joshua Ladau, Tamsen Polley, Lyndsey Jean Marsh, Negeen Najafi, Pramod Pandey, Latika Singh, Robert Kinley, Joan King Salwen, Emiley Eloe-Fadrosh, Ermias Kebreab, Matthias Hess

**Affiliations:** 1 Department of Animal Science, University of California, 2251 Meyer Hall, Davis, CA 95616 USA; 2 Department of Energy Joint Genome Institute, 2800 Mitchell Drive, Walnut Creek, CA 94598 USA; 3 Department of Population Health and Reproduction, School of Veterinary Medicine, One Shields Avenue, Davis, CA 95616 USA; 4 Department of Earth System Science, Stanford University, 450 Serra Mall, Stanford, CA 94305 USA; 5 Agriculture and Food, Commonwealth Scientific and Industrial Research Organisation (CSIRO), Building 145 James Cook Drive, James Cook University, Townsville, QLD 4811 Australia

**Keywords:** 16S rRNA community profiling, *Asparagopsis taxiformis*, Feed supplementation, Greenhouse gas mitigation, In-vitro rumen fermentation, Macroalgae, Rumen microbiome

## Abstract

**Background:**

Recent studies using batch-fermentation suggest that the red macroalgae *Asparagopsis taxiformis* has the potential to reduce methane (CH_4_) production from beef cattle by up to ~ 99% when added to Rhodes grass hay; a common feed in the Australian beef industry. These experiments have shown significant reductions in CH_4_ without compromising other fermentation parameters (i.e. volatile fatty acid production) with *A. taxiformis* organic matter (OM) inclusion rates of up to 5%. In the study presented here, *A. taxiformis* was evaluated for its ability to reduce methane production from dairy cattle fed a mixed ration widely utilized in California, the largest milk producing state in the US.

**Results:**

Fermentation in a semi-continuous in-vitro rumen system suggests that *A. taxiformis* can reduce methane production from enteric fermentation in dairy cattle by 95% when added at a 5% OM inclusion rate without any obvious negative impacts on volatile fatty acid production. High-throughput 16S ribosomal RNA (rRNA) gene amplicon sequencing showed that seaweed amendment effects rumen microbiome consistent with the Anna Karenina hypothesis, with increased β-diversity, over time scales of approximately 3 days. The relative abundance of methanogens in the fermentation vessels amended with *A. taxiformis* decreased significantly compared to control vessels, but this reduction in methanogen abundance was only significant when averaged over the course of the experiment. Alternatively, significant reductions of CH_4_ in the *A. taxiformis* amended vessels was measured in the early stages of the experiment. This suggests that *A. taxiformis* has an immediate effect on the metabolic functionality of rumen methanogens whereas its impact on microbiome assemblage, specifically methanogen abundance, is delayed.

**Conclusions:**

The methane reducing effect of *A. taxiformis* during rumen fermentation makes this macroalgae a promising candidate as a biotic methane mitigation strategy for dairy cattle. But its effect in-vivo (i.e. in dairy cattle) remains to be investigated in animal trials. Furthermore, to obtain a holistic understanding of the biochemistry responsible for the significant reduction of methane, gene expression profiles of the rumen microbiome and the host animal are warranted.

**Electronic supplementary material:**

The online version of this article (10.1186/s42523-019-0004-4) contains supplementary material, which is available to authorized users.

## Background

Methane (CH_4_) is a major greenhouse gas with a global warming potential 28-fold greater than that of carbon dioxide (CO_2_) on a 100-year scale [1] and it accounts for approximately 11% of the greenhouse gas (GHG) emissions in the US [2]. Enteric fermentation from ruminant animals alone accounts for approximately 25% of the total CH_4_ emissions in the US; representing the largest anthropogenic source of CH_4_ [3]. Increasing emphasis on reducing GHG emissions from the livestock industry requires advanced methods for reducing and controlling CH_4_ production. Identifying efficient strategies to lower enteric CH_4_ production could result in a significantly reduced carbon footprint from animal production and provide the cattle industry with a way to meet legislative requirements; calling for a reduction of CH_4_ emission of ~ 40% by 2030.

The biological production of CH_4_ in the rumen is the product of symbiotic relationships between fiber degrading bacteria, hydrogen (H_2_) producing protozoa and methanogenic archaea [4, 5]. Besides being converted into CH_4_, metabolic H_2_ may also be incorporated into volatile fatty acids (VFA), such as acetate, propionate, and butyrate which are then used as energy by the ruminant animal. Theoretically, inhibiting methanogenesis could free molecular H_2_ for use in pathways that produce metabolites (i.e. VFAs) that are more favorable to the host animal, thus creating potential for increased feed efficiency. Since production of enteric CH_4_ can account for up to 12% of the total energy consumed by the animal [6, 7] even a small reduction of CH_4_ production and redirection of carbon molecules into more favorable compounds has the potential to result in significantly more economically and ecologically sustainable production practices in the ruminant industry.

Extensive research has been performed on the effectiveness of feed supplements to reduce enteric CH_4_ emissions through inhibition of microbial methanogenesis within the rumen system [8]. Results have been reported for a number of feed supplements including inhibitors, ionophores, electron receptors, plant bioactive compounds, dietary lipids, exogenous enzymes, and direct-fed microbials indicating reductions on CH_4_ production [9]. While several of these compounds have been shown to inhibit ruminal methanogenesis, some have been shown to decrease VFA production [10], which decreases overall nutrient availability to the animal, and is therefore a non-desirable side effect.

Algae are a stable component of the human diet in some cultures [11] and have also been used as feed for agricultural products such as abalone [12] and shrimp [13]. The ability of algae to promote well-being and health is mediated to a great extent by highly bioactive secondary metabolites [14–16] that are synthesized by some algal species [17]. Additionally, some of the brown and red macroalgae have shown to inhibit microbial methanogenesis when tested in-vitro [18] and a similar response of the animal microbiome has been proposed. These findings suggest that macroalgae could promote higher growth rates and feed conversion efficiencies in ruminants [19, 20]. Macroalgal supplementation shows great promise as a CH_4_ mitigation strategy during enteric fermentation [10, 18, 21, 22]. Macroalgae feed supplementation may therefore be an effective strategy to simultaneously improve profitability and sustainability of cattle operations.

Various types of algae have antibacterial, antiviral, antioxidant, anti-inflammatory, and anti-carcinogenic properties [23–26]. Most recently, macroalgae has been tested in-vitro and in-vivo to determine if there are anti-methanogenic properties within selected types of macroalgae. *Asparagopsis taxiformis*, a red macroalgae, seems to be the most effective species of macroalgae to reduce methane production.

A recent study identified *Asparagopsis taxiformis*, as a highly efficient feed supplement for CH_4_ mitigation during enteric fermentation [18]. In this work, the effect of a large variety of macroalgal species including: freshwater, green, red, and brown algae on CH_4_ production during in-vitro incubation was compared. Results showed *A. taxiformis* amendment yielded the most significant reduction (~ 98.9%) of CH_4_ production. Moreover, *A. taxiformis* supplementation at inclusion rates up to 5% organic matter (OM) revealed methane reduction by 99% without significant negative impact on VFA profiles and OM digestibility, in-vitro [10]. Furthermore, *A. taxiformis* was determined to contain an abundance of anti-methanogenic compounds including: bromoform, dibromocholoromethane, bromochloroacetic acid, dibromoacetic acid, and dichloromethane [27]. Bromoform, a halomethane, is the most abundant antimethanogenic compound found in *A. taxiformis*, and has been shown to inhibit enzymatic activities by binding to vitamin B_12_ [28]; which chemically resembles coenzyme F430 a cofactor needed for methanogenesis [29]. Additionally, it has been shown that *A. taxiformis* reduces CH_4_ production during enteric fermentation more effectively than highly concentrated halogenated methane analogs [30]. It has been suggested that the increased efficiency of *A. taxiformis* may be due to multiple antimethanogenic bioactives working synergistically [30]. While it is clear that *A. taxiformis* contains antimethanogenic compounds, actual concentrations of these compounds seem to vary and what causes these variations remain unclear.

In the work presented here, we studied the effect of *A. taxiformis* (5% OM inclusion rate) on the rumen microbiome assemblage and function during in-vitro fermentation over the duration of four days. A better understanding of how this macroalgae affects CH_4_ production from dairy cows fed a diet commonly used in California should provide insight into the value of an *A. taxiformis*-based CH_4_ mitigation strategy for the dairy industry in California. Additionally, high-throughput 16S rRNA amplicon sequencing was used to provide new insights of the affects of *A. taxiformis* supplementation on the rumen microbiome assemblage. To our knowledge this is the first time that this highly efficient procedure was employed to dissect the changes of the rumen microbiome in dairy cattle in response to *A. taxiformis* as a feed supplement and CH_4_ mitigator.

## Results

### In-vitro standard measurements remained stable throughout the experiment

**Fig. 1:**
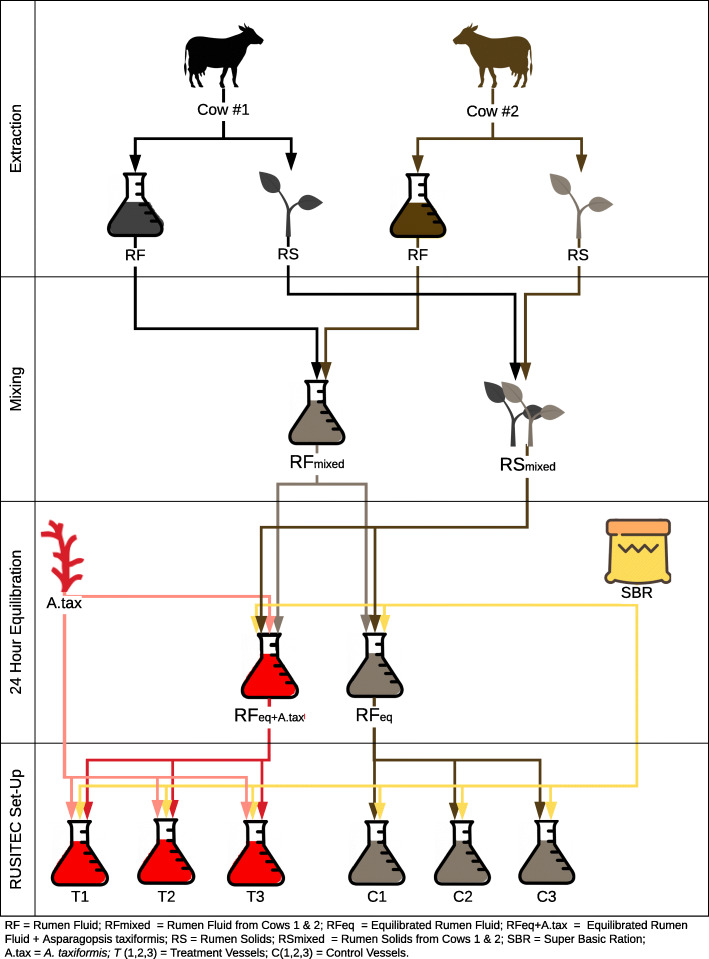
In-vitro rumen system set-up. Extraction: Rumen fluid and rumen solids were collected from 2 dairy cows. Mixing: Rumen fluid was homogeneously mixed and rumen solids were homogeneously mixed. After mixing, rumen fluid was separated into two Erlenmeyer flasks, where treatment was then assigned. 24 Hour Equilibration: The control flask received 30 g of mixed rumen solids and 30 g of SBR and the treatment flask received 30 g of mixed rumen solids, 30 g of SBR, and 1.5 g of *A. taxiformis*. After each flask received their treatment, the 24 h equilibration period began. After the equilibration period, each flask was then divided into 3 vessels, then fed their respective treatments (control = 10 g SBR/vessel, treatment = 10 g SBR/vessel & .2 g *A. taxiformis*)

Rumen fluid and rumen solids were collected from two fistulated dairy cattle. Rumen contents were homogenized and equilibrated for 24 h and subsequently inoculated into the artificial gut system following the experimental design outlined in Fig. 1. Temperature, pH, and mV remained relatively constant (37 °C ± 2, 6.8 pH ±0.03, 21 mV ±3) throughout the entire experiment and between individual vessels.

### *A. taxiformis* contains an elevated mineral profile but less organic matter compared to SBR

A higher OM content for SBR was found (92.8% DM) when compared to *A. taxiformis* (53% DM). Crude protein amounts were relatively similar for SBR (20% DM) and *A. taxiformis* (17.8% DM). Neutral detergent fiber composition of SBR and *A. taxiformis* were also similar with 38.1 and 36.9% DM, respectively. Differences in starch content between SBR and *A. taxiformis* were prominent with 12.6 and 0.7% DM, respectively. Lignin content for SBR was determined with 6% DM and 4.4% DM for *A. taxiformis*. Total digestible nutrient content (TDN) for *A. taxiformis* was approximately half (33.8% DM) of the TDN determined for SBR (66.2% DM). *Asparagopsis taxiformis* contained elevated mineral profiles compared to SBR. More specifically, *A. taxiformis* exhibited higher calcium, sodium, magnesium, iron, and manganese concentrations. Zinc was present at 23.7 ppm in both SBR and *A. taxiformis*. The detailed composition of SBR and *A. taxiformis* is shown in Table 1.

**Table 1:** Composition of SBR and *Asparagopsis taxiformis*

	SBR^a)^	*A. taxiformis*
Chemical Composition		
% Dry matter		
Organic matter	92.8	53
Crude protein	20.0	17.8
Neutral detergent fiber	38.1	36.9
Acid detergent fiber	27.3	11.6
Starch	12.6	0.7
Fat	2.7	0.4
Total digestible nutrients	66.2	33.8
Lignin	6	4.4
Calcium	0.9	3.8
Phosphorus	0.4	0.2
Sodium	0.1	6.6
Magnesium	0.5	0.8
Parts per million		
Iron	632.7	6241
Manganese	41.7	112.7
Zinc	23.7	23.7
Copper	11	8.7

^a)^Super basic ration

### *A. taxiformis* decreases methane production and increases propionate:Acetate ratio

**Fig. 2:**
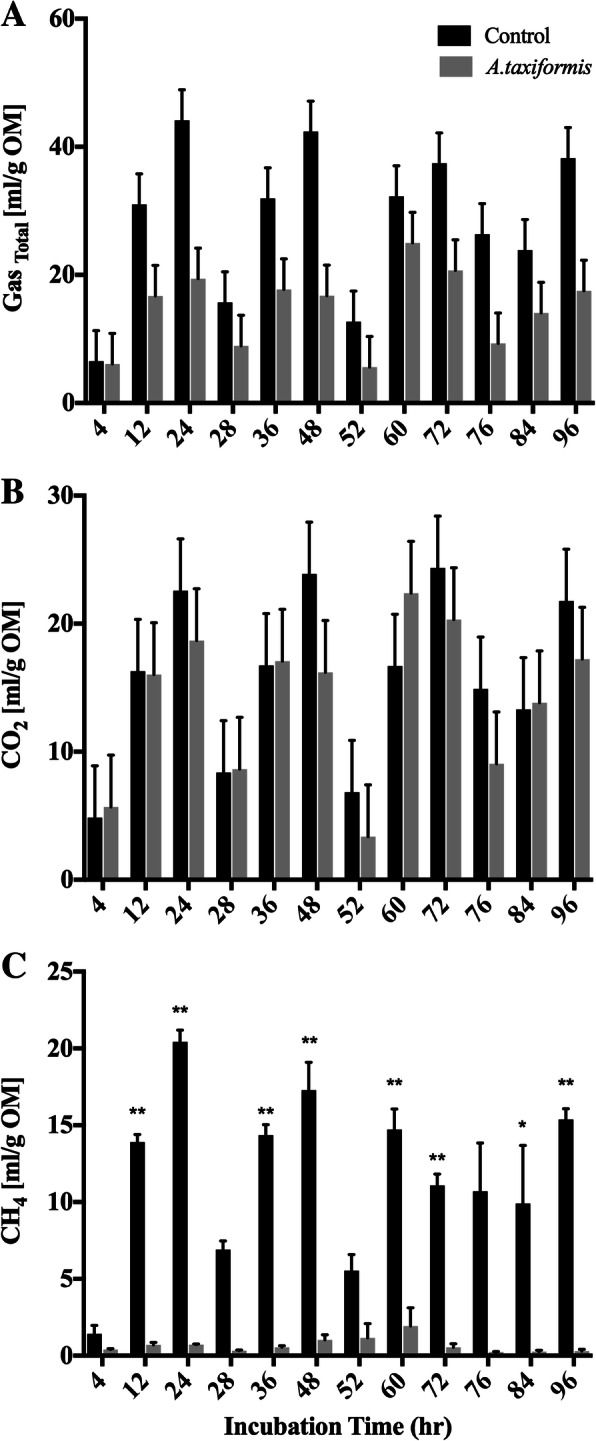
Total gas, CH_4_, and CO_2_ production during in-vitro fermentation. Production of total gas, CH_4_ and CO_2_ [ml/(g OM)] from vessels without (*n* = 3) and with (n = 3) *A. taxiformis* as additive at 4, 12, and 24 h over the course of the experiment. **a** Total gas production; **b** CH_4_ production; **c** CO_2_ production. Measurement were performed in triplicates. “**” indicates significant difference (*p* value ≤0.05), “*” indicates trend toward significance (0.05 > *p* value ≤0.1)

Total gas production (TGP) and CH_4_ production were significantly affected by the inclusion of *A. taxiformis* (*p* < 0.05, Table 2). Average total gas production for the *A. taxiformis* treatment group was 14.81 ml/(g OM) whereas the control group was 28.54 ml/(g OM), representing a 51.8% reduction in TGP with *A. taxiformis*. Average CH_4_ production for the *A. taxiformis* treatment group was 0.59 ml/(g OM), whereas the control group produced 12.08 ml/(g OM), representing a 95% reduction of CH_4_ being synthesized. No significant difference was found in CO_2_ production between the *A. taxiformis* treatment and the control groups. Figure 2 illustrates how total gas (i.e. CH_4_ and CO_2_) was affected over the duration of the experiment. It appears that *A. taxiformis* is effective at reducing TGP and CH_4_ almost immediately, beginning at 12 h after the beginning of the experiment, and continues to inhibit CH_4_ production over 24 h just prior to when new bioactive is provided during the feeding process (at 24 h 48 h, and 72 h). Inhibition of methanogenesis was also measured just prior to the termination of the experiment (96 h).

**Table 2:** Effects of *A. taxiformis* on total gas production and total volatile fatty acid production

	Control	*A. taxiformis*	Standard error	*p* value
Gas Production [ml/(g OM)]				
CH_4_	12.08	0.59	0.59	< 0.0001
CO_2_	15.67	14.24	3.82	0.73
Total Volume	28.54	14.81	3.85	0.02
Volatile Fatty Acid Production [ppm]				
Total VFA	2332.52	2105.11	269.2	0.45
Acetate	1056.99	856.77	135.08	0.21
Propionate	481.12	490.54	58.36	0.88
Propionate:Acetate^a)^	0.48	0.6	0.01	< 0.001
Butyrate	394.35	423.01	53.55	0.62
Isobutyrate	84.81	79.83	4.32	0.31
Valerate	212.79	168.72	16.99	0.06
Isovalerate	102.44	86.21	14.49	0.33

^a)^reported as a ratio of respective VFAs

**Fig. 3:**
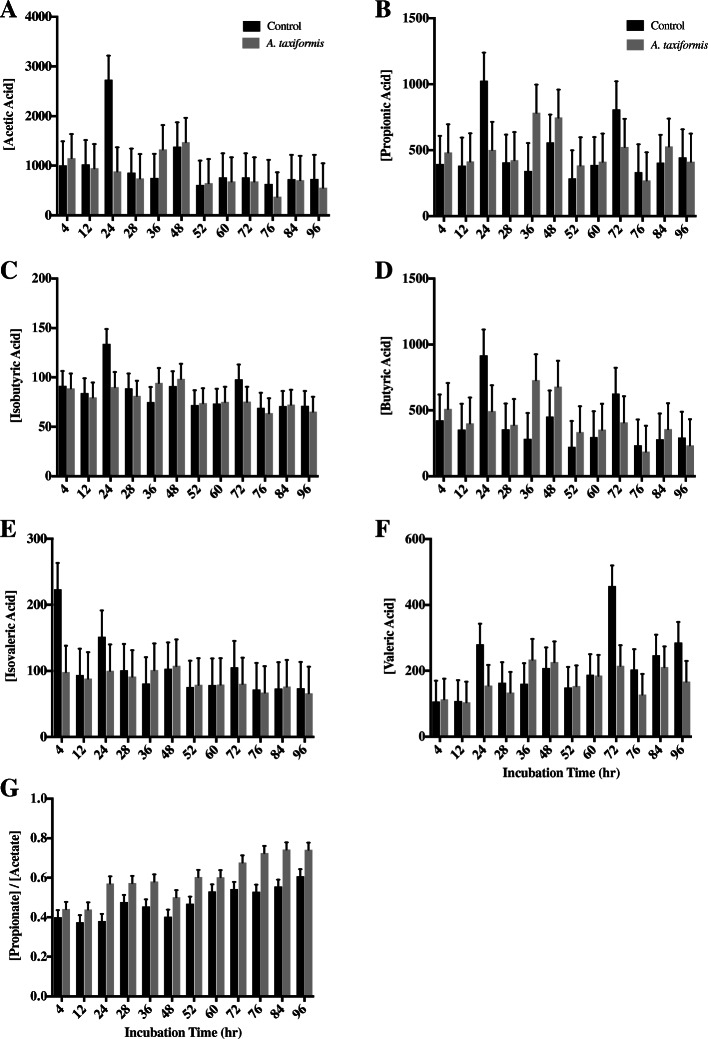
Volatile fatty acid production during in-vitro fermentation. Volatile fatty acid concentrations [ppm] of fermentation fluid of vessels without (n = 3) and with (n = 3) *A. taxiformis* as additive, determined 4, 12, and 24 h after feeding over 4 days. **a** Acetic acid; **b** Propionic acid; **c** Isobutyric acid; **d** Butyric acid; **e** Isovaleric acid **f** Valeric acid; **g** Propionate/Acetate Ratio. Measurement were performed in triplicates

Slightly higher total VFA concentrations were recorded for the control group when compared to the *A. taxiformis* treatment group [2332.52 ppm vs. 2105.11 ppm ± 269.20 ppm respectively (means ± SE)], however this difference was not statistically significant (*p* = 0.45, Table 2). Additionally, no significant differences were found when comparing concentrations of acetate, propionate, butyrate, isobutyrate, valerate, and isovalerate (Table 2) between control and *A. taxiformis* treatment group. Although, valerate was not found to be statistically different between groups (*p* < 0.05), it was observed that the *A. taxiformis* treatment group tended to have lowered concentrations of valerate when compared to the control group (*p* = 0.06). Statistical differences were found between groups when comparing the propionate:acetate ratio, with a higher proportion of propionate to acetate within the *A. taxiformis* treatment groups (*p* = 0.001). Differences observed at each timepoint between control and *A. taxiformis* treatment groups were determined to be not significant (Fig. 3).

### Sequencing and quality filtering

A total of 1,251,439 reads were generated from a total of 77 samples, with a mean (± SD) of 16,275 (±1879) reads per sample. After quality filtering, 757,325 (60.5%) high quality sequences remained. Operational taxonomic units (OTU) based analysis (at 97% sequence identity) revealed 32,225 unique OTUs across all samples. Singletons contributed 23,043 (3%) unique reads to the total filtered read count, and were removed prior further analysis. The mean Goods’ coverage for all samples was 88 ± 3%, suggesting that the sequencing effort recovered a large proportion of the microbial diversity in each of the samples under investigation. Distribution of the number of OTUs among each condition and time point during the experiment can be found in Additional file 1: Table S1.

### α-Diversity measurements show microbial communities diverged slightly over the course of the experiment

The microbial communities of the control and *A. taxiformis* amended vessels were compared at each incubation time. Significant differences in the microbial community between the two conditions appeared transiently at only two time points, the 12 h time point on the first day of the experiment and again at the 24 h time point on the fourth day (96 h after the start of the experiment, AMOVA, *p* ≤ 0.02, and *p* ≤ 0.04 respectively). Comparison of the microbial communities from the start and end of the experiment within each group suggested that the microbial communities changed over the course of the experiment (AMOVA, *p* ≤ 0.06 and *p* ≤ 0.05, treatment and control respectively). The divergence of the microbial communities throughout the experiment was visualized by Principal Coordinate Analysis (PCoA) and is illustrated in Additional file 1: Figure S2. The communities associated with treatment and control are very similar at the beginning but started to diverge immediately after the initiation of the experiment (4 h). While the diverging trajectory becomes more apparent throughout the experiment (i.e, 96 h), the first two axes of the PCoA plot account for a low fraction (13.5%) of the total variation that is observed between the samples, which coincides with the observation that the communities associated with the two vessel groups were largely similar.

### Microbial communities respond to *A. taxiformis* as a stressor, but recover quickly

**Fig. 4:**
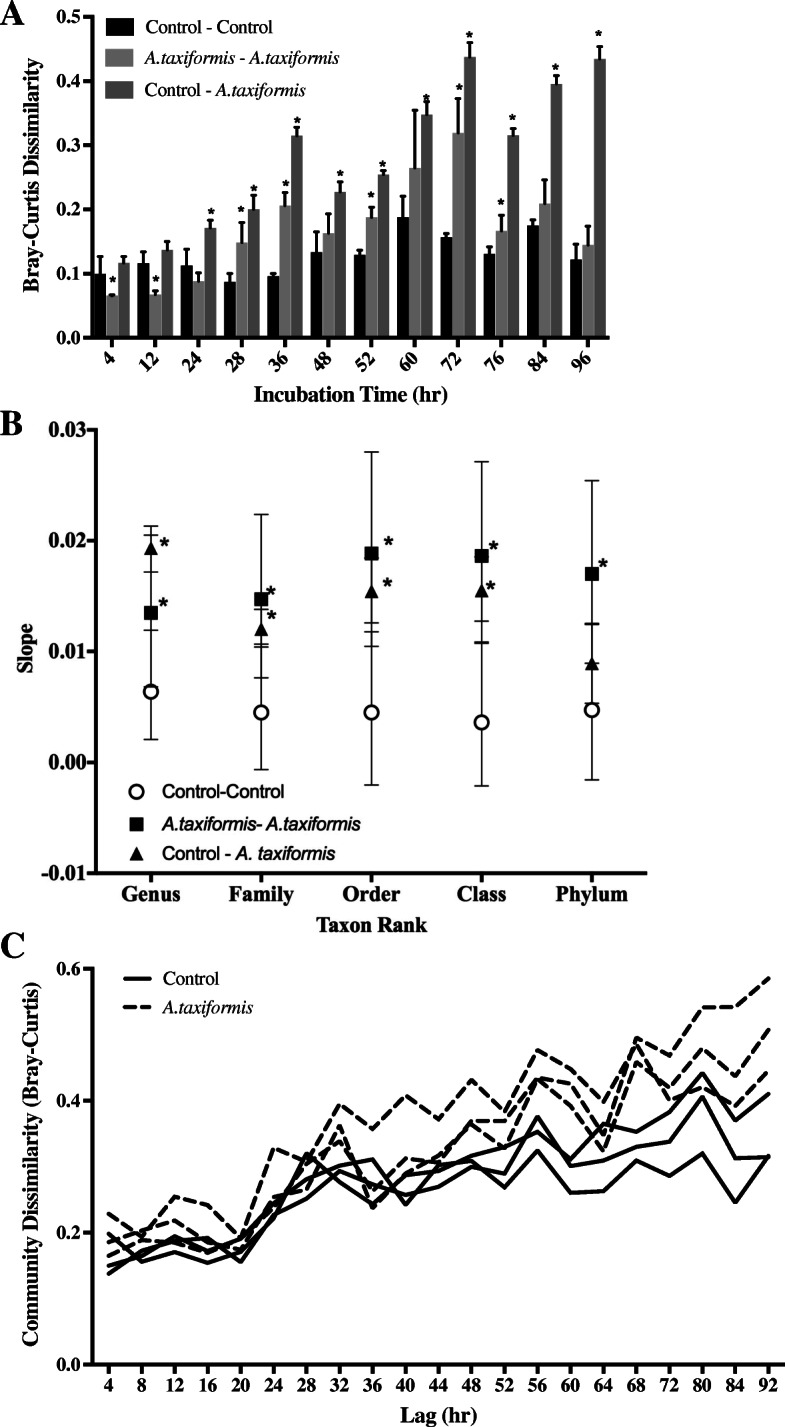
Effects of seaweed amendments on composition of in-vitro rumen microbiome. **a** Genus-level β-diversity between pairs of vessels throughout the duration of the experiment. **b** β-diversity across multiple taxonomic groups measured between pairs of samples versus sampling time for each of the 6 vessels. 95% bootstrap confidence intervals are shown. Regression slopes identified as significant (*p* < 0.001) by a permutation test are indicated with an asterisk. **c** Genus-level β-diversity within individual vessels across different sampling times

Although the effects of seaweed amendments on methane production were immediate (≤ 12 h), amendments may also affect microbial populations on a longer time scale. Over the duration of the experiment, β-diversity between pairs of control vessels remained constant (permutation test for non-zero slope: *p* > 0.001). In contrast, β-diversity between pairs of treatment vessels and between treatment and control vessels gradually changed. More specifically, β-diversity between treatment vessels increased and then decreased, with highest difference measured at ~ 72 h after the start of the experiment, while β-diversity between treatment and control vessels increased essentially monotonically until the end of the experiment (Fig. 4a; permutation test for non-zero slope: *p* < 0.001). These slow shifts in community composition were evident regardless of the taxonomic level at which β-diversity was considered, including at coarse taxonomic resolutions (Fig. 4b). Examination of the genus-level β-diversity within vessels across different time lags also indicated that the microbial communities continued to shift throughout the duration of the experiment (Fig. 4c). Essentially, sample pairs collected at more distant times were on average more dissimilar than those collected at similar times. This trend was most pronounced for pairs of samples that had seaweed amendments.

### Average methanogen abundance decreased, but not in concert with methane reduction

**Fig. 5:**
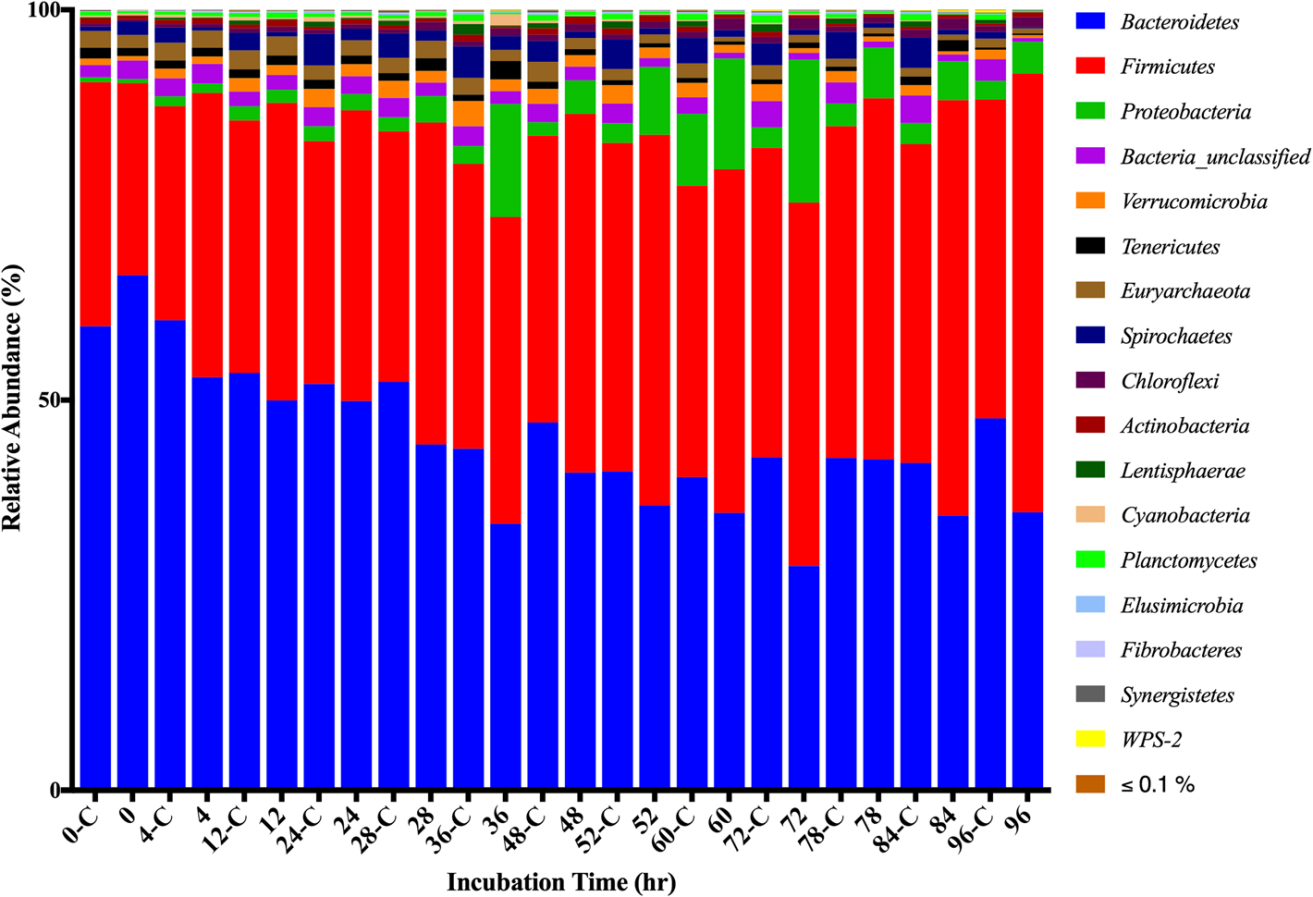
Relative abundance of phyla during in-vitro fermentation. Fermentations were performed in three in-vitro vessels (n = 3). Incubation times annotated with “C” represent control conditions

**Fig. 6:**
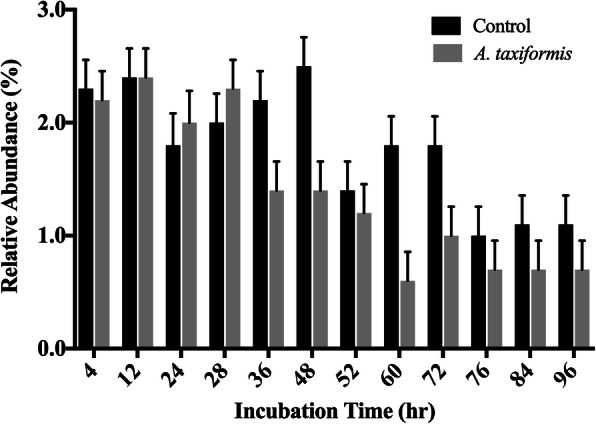
Relative abundance of Euryarchaeota during in-vitro fermentation. Fermentations were performed in three in-vitro vessels (n = 3). Error bars indicate standard error of the mean

Across all samples, one archaeal and 21 bacterial phyla were identified. The ten most abundant phyla recruited > 98% of the reads generated from the microbial communities of both the control and *A. taxiformis* amended vessels (Fig. 5). Microbiomes throughout the experiment, regardless of experimental condition or time, were dominated by *Bacteroidetes*, *Firmicutes*, and *Proteobacteria*. The *Bacteroidetes:Firmicutes* ratio decreased in both conditions over the course of the experiment, suggesting influence due to the experimental system (Fig. 5). With the drastic decrease in CH_4_ in mind, the differences between the two groups were investigated at a finer resolution by exploring the abundance dynamics of the Archaeal phylum Euryarchaeota, which include the methanogenic Archaea. Based on the 16S rRNA gene profiles, five genera of methanogenic Archaea were identified in all stages of the experiment. The five genera: *Methanobrevibacter*, *Methanosphaera*, *vadin CA11* of the *Methanomassiliicoccacaea* family, *Methanoplanus* and *Methanimicrococcus* accounted for all reads recruited by the Euryarchaeota. *Methanobrevibacter* and *Methanosphaera* accounted for > 99% of the reads assigned to methanogens. While CH_4_ production decreased in the *A. taxiformis* amended vessels 12 h after the first feeding event, abundance of methanogenic Archaea in the two conditions did not differ significantly at individual time points (Fig. 6). However, the average relative abundance of Euryarchaeota over the duration of the experiment were lower in the *A. taxiformis* amended vessels compared to control vessels (1.38 and 1.79% respectively, *p* ≤ 0.03).

## Discussion

A significant reduction in CH_4_ production was found when evaluating the effects of *A. taxiformis* on ruminal fermentation characteristics, in-vitro*,* at a 5% OM inclusion rate. Results from the overall experiment show an approximate decrease in TGP by ~ 50% and in CH_4_ production by ~ 95%, which is similar to multiple studies conducted on the effects of *A. taxiformis,* both in-vivo and in-vitro [10, 18, 30, 31].

Carbon dioxide production remained similar between the control and *A. taxiformis* amended vessels. Comparison of total and individual VFA between vessels did not suggest any difference in VFA production at any specific time point with the 5% OM inclusion rate. A significant reduction of CH_4_ was measured 12 h after *A. taxiformis* amendment (Fig. 2), while CO_2_ production and VFAs profiles remained unchanged throughout the fermentation process (Figs. 2 and 3). This suggests that the amendment of SBR supplemented with *A. taxiformis*, inhibits methanogenesis but not CO_2_ production, which is often used as a measurement for microbial growth. This targeted effect on a specific metabolic function, and hence a functional group within the microbiome, was also elucidated from the 16S rRNA profiles of the in-vitro rumen system. he overall assemblages of the microbiome associated with the treatment and control fermentation vessels remained rather similar throughout the duration of the fermentation process (Fig. 5). Changes in the relative abundance of members belonging to the Euryarchaeota, the taxonomic group that encompasses the main rumen methanogens, could be observed as early as 36 h after the initiation of the experiment. Although a semi-continuous batch fermentation system, as utilized for this study, is capable of maintaining more rumen like conditions, mainly through maintaining adequate pH and nutrient levels, when compared to a simple batch fermentation process, a wash-out of the more sensitive rumen microbes (i.e. protozoa) is inevitable [32]. It is well known that there is a mutualistic relationship between protozoa and methanogens [33, 34], and it has been shown before that the removal of rumen protozoa results in a reduction of the methanogen population and methanogenesis during enteric fermentation [35, 36]. Hence, the decrease in relative abundance of Euryarchaeota observed for the control vessels at later time points of the experiment is most likely an artifact caused by the inability of the in-vitro systems to maintain protists over an extended period of time.

### Propionate:Acetate ratio increased in treatment vessels

Over the course of the experiment, the propionate:acetate ratio increased (*p* < 0.001) in treatment vs control groups. The first step of the formation of acetate in the rumen releases metabolic hydrogen which acts as a hydrogen donor to methanogenic archaea and therefore facilitates the production of CH_4_ in the rumen [37]. In contrast, propionate acts as a competing hydrogen sink [4, 38]. The increased propionate:acetate ratio suggest that hydrogen is, at least in some part, being redistributed to propionate, which may help explain a portion of the methane reduction seen here. In the context of dairy cattle and milk production, the increased propionate:acetate ratio seen in vessels amended with *A. taxiformis* may forecast an altered milk composition in-vivo. A decreased propionate:acetate ratio is associated with increased milk fat, and total milk yield is positively associated with butyrate and propionate in the rumen [39]. Under this paradigm, *A. taxiformis* supplementation has the potential to increase total milk yield, however may also negatively impact milk fat content.

### Microbial communities overcame the stress of treatment

We observed that *A. taxiformis* has affects consistent with the Anna Karenina Hypothesis, which posits that disturbances act to increase differentiation of microbial communities [40]. Specifically, we found that communities in treatment vessels differentiated increasingly from each other up to hour 72, after which they reconverged (Fig. 4a). This finding suggests that, the rumen microbial community undergoes changes that are both slow and variable in response to *A. taxiformis*. However, these changes do not appear to be associated with variability in reduction of gas production. While *A. taxiformis* may pose an initial stress on the rumen microbial community, measured by the increased differentiation between treatment vessels, the β-diversity between communities in amended vessels stabilized after only 72 h under recurrent daily stress (feeding).

### *A. taxiformis* is a potential mineral supplement

Nutritional analysis of *A. taxiformis* revealed that *A. taxiformis* has high levels of important minerals including calcium, sodium, iron, and manganese (Table 2) suggesting that in addition to its methane reduction potential, *A. taxiformis* may also be used to increase mineral availability to basic rations. In-vivo studies directed towards monitoring mineral transfer from feed into product should be conducted next to facilitate a better understanding of whether or not minerals, or other compounds, present in seaweed can be found in milk or meat of the consuming animals. While halogen compounds have been reported as important players in the bioactive process of methane reduction, previous studies using seaweed as a feed supplement found that iodine, which is abundant in brown algae, is found in the milk of cows to which it is fed [41].

## Conclusions

The methane reducing effect of *A. taxiformis* during rumen fermentation of feed makes this macroalgae a promising candidate as a biotic methane mitigation strategy for California dairy producers. The organic matter inclusion required to achieve such a drastic decrease in methane is low enough to be practically incorporated in the rations of average dairy operations. Significant limitations to the implementation of *A. taxiformis,* and potentially other algae, include the infrastructure and capital necessary to make these products commercially available and affordable. Furthermore, our understanding of the host microbe interactions during seaweed supplementation are limited. In order to obtain a holistic understanding of the biochemistry responsible for the significant reduction of methane, and its potential long-term impact on ruminants, gene expression profiles of the rumen microbiome and the host animal are warranted.

## Methods

### Animals, diets and rumen content collection

All animal procedures were performed in accordance with the Institution of Animal Care and Use Committee (IACUC) at University of California, Davis under protocol number 19263. Rumen content was collected from two rumen fistulated cows, one Jersey and one Holstein, housed at the UC Davis Dairy Unit. Animals were fed a dry cow total mixed ration (50% wheat hay, 25% alfalfa hay/manger cleanings, 21.4% almond hulls, and 3.6% mineral pellet (Table 1). Three liters of rumen fluid and 60 g of rumen solids were collected 90 min after morning feeding. Rumen content was collected via transphonation using a perforated PVC pipe, 500 mL syringe, and Tygon tubing (Saint-Gobain North America, PA, USA). Fluid was strained through a colander and 4 layers of cheesecloth into two 4 L pre-warmed, vacuum insulated containers and transported to the laboratory.

### In-vitro feed and feed additive composition and collection

Due to its wide utilization in the dairy industry for cows during lactation, super basic ration (SBR) was used as feed in the in-vitro experiment. SBR was composed of 70% alfalfa pellets, 15% rolled corn, and 15% dried distillers’ grains (Table 3). Individual components were dried at 55 °C for 72 h, ground through a 2 mm Wiley Mill (Thomas Scientific, Swedesboro, NJ) and manually mixed. *Asparagopsis taxiformis* used as feed additive was provided in kind from the Commonwealth Scientific and Industrial Research Organization (CSIRO) Australia. The macroalgae was in its filamentous gametophyte phase when collected near Humpy Island, Keppel Bay, QLD (23^o^13'01"S, 150^o^54'01"E) by MACRO (Center for Macroalgal Resources and Biotechnology) of James Cook University (JCU) in Townsville, QLD. The collected biomass was frozen and stored at − 15 °C then shipped to Forager Food Co. in Red Hills, Tasmania, AUS, where it was freeze dried and milled (2–3 mm) to ensure a uniform product. Chemical composition of SBR and of *A. taxiformis* were analyzed at Cumberland Analytical Services (Waynesboro, PA).

**Table 3:** Composition of dry cow diet and super basic ration (SBR)

Dry Cow Diet		SBR	
Ingredient			
Alfalfa	25%	Alfalfa	70%
Wheat	50%	Dried distillers grain	15%
Almond hulls	21.40%	Rolled corn	15%
Mineral pellets	3.60%		

### Engineered (in-vitro) rumen system

An advanced semi-continuous fermentation system, with six 1 L vessels with peristaltic agitation, based on the rumen simulation technique (RUSITEC) developed by Czerkawski and Breckenridge [42] was used to simulate the rumen in the laboratory.

### Experimental design

Equilibration (Day 0): Temperature, pH and conductivity of the rumen fluid and solids were recorded using a mobile probe (Extech Instruments, Nashua, NH). Rumen fluid, 3 L, from each cow were combined with 2 L of artificial saliva buffer [43] homogenized and then split into two 3 L aliquots. Rumen solids, 15 g, from each animal were sealed in Ankom concentration bags (Ankom, Macedon, NY) and added to each equilibration vessel (30 g of rumen solids per vessel total). Three concentrate bags containing 10 g of SBR each were added to each vessel. One of the equilibration vessels was amended with 5% (*w*/w) of *A. taxiformis* 24 h prior to the start of the experiment (Fig. 1). Content of the equilibration vessel without *A. taxiformis* was used to inoculate control vessels of the in-vitro system, whereas content of the equilibration vessel with *A. taxiformis* was used to inoculate the treatment vessels (Fig. 1). SBR was ground in a 2 mm Wiley Mill before being added to each concentrate bag to increase substrate availability and therefore producing similar particle sizes that which the mastication function in-vivo provides to the animal. The two vessels were then placed in a 39 °C water bath and stirred with a magnetic stir bar for a 24 h equilibration period.

*Fermentation* (Days 1–4): After 24 h of equilibration, temperature, pH, and conductivity of the rumen fluid were recorded to determine stability of the vessels and their content. Each of the 6 in-vitro rumen vessels were randomly designated as either treatment or control vessel and filled with 750 mL of the corresponding fluid from the equilibration vessels. Location of the vessels within the in-vitro platform were randomly allocated.

Each vessel received one concentrate bag of SBR from its respective equilibration vessel and one new concentrate bag. Control concentrate bags contained 10 g SBR. Treatment concentrate bags contained 10 g SBR plus 5% (OM) *A. taxiformis*. To simulate rumen retention time, each of the feedbags were incubated in the allocated fermentation vessel for 48 h. Temperature, pH, and conductivity were measured every 24 h prior to exchanging one of the concentrate bags (feeding). After each feeding, all vessels were flushed with N_2_ to maintain anaerobic conditions within the reactors. Individual reactor vessels of the artificial rumen system were connected to a reservoir containing artificial saliva buffer. A peristaltic pump delivered 0.39 mL/min of buffer to each vessel throughout the course of the experiment. Gas bags (Restek, USA) and overflow vessel were used to continuously collect generated gas and effluent fluid. Effluent vessels were chilled with ice to mitigate residual microbial activity. An outline of the experimental set-up and the preparation of the treatment and control vessels is provided in Fig. 1.

### Sample collection and analysis

Liquid and gas sample collections took place at 3 time points every 24 h for 4 days. Time point intervals were 4, 12, and 24 h post-feeding each day. Fluid samples were collected in 1.5 mL tubes, flash frozen in liquid nitrogen, and stored at − 20 °C until processed. Gas bags were collected at each time series interval for analysis of total gas production, CO_2_ and CH_4_ concentrations. Gas volume was measured with a milligas flow meter (Ritter, Germany) by manual expulsion of the collection bag.

### Volatile fatty acid and greenhouse gas analysis

To determine VFA profiles, Gas Chromatography-Flame Ionization detection (GC-FID) was used. Fermentation fluid was prepared for VFA analysis by mixing with 1/5th volume 25% metaphosphoric acid, and centrifugation. Supernatant was filtered through a 0.22 μm filter and stored in amber autosampler vials at 4 °C until analysis. The GC conditions were as follows: analytical column RESTEK Rxi® – 5 ms (30 m × 0.25 mm I.D. × 0.25 μm) film thickness; the oven temperature was set to 80 °C for 0.50 min, and followed by a 20 °C/min ramp rate until 200 °C, holding the final temperature for 2 min; carrier gas was high purity helium at a flow rate of 2.0 mL/min, and the FID was held at 250 °C. A 1 μL sample was injected through Split/Splitless Injectors (SSL), with an injector base temperature set at 250 °C. Split flow and split ratio were programmed at 200 and 100 mL/min respectively. To develop calibration curves, certified reference standards (RESTEK, Bellefonte, PA) were used. All analyses were performed using a Thermo TriPlus Autosampler and Thermo Trace GC Ultra (Thermo Electron Corporation, Rodano Milan, Italy).

Methane and CO_2_ were measured using an SRI Gas Chromatograph (8610C, SRI, Torrance, CA) fitted with a 3’× 1/8″ stainless steel Haysep D column and a flame ionization detector with methanizer (FID-met). The oven temperature was held at 90 °C for 5 min. Carrier gas was high purity hydrogen at a flow rate of 30 ml/min. The FID was held at 300 °C. A 1 mL sample was injected directly onto the column. Calibration curves were developed with an Airgas certified CH_4_ and CO_2_ standard (Airgas, USA).

### DNA extraction

DNA extraction was performed using the FastDNA SPIN Kit for Soil (MP Biomedicals, Solon, OH) with ~ 500 mg of sample according to the manufacturer’s protocol. DNA was subsequently purified with a Monarch® PCR & DNA Cleanup Kit (New England Biolabs, Ipswich, MA) following the manufacturer’s instructions. Extracted DNA was stored at − 20 °C until subsequent PCR amplification and amplicon sequencing.

### PCR amplification, library preparation, and sequencing

The V4-V5 hypervariable region of the 16S rRNA gene was sequenced on Illumina’s MiSeq platform using the 515yF (3′-GTG YCA GCM GCC GCG GTA A-5′) and 926pfR (3’-CCG YCA ATT YMT TTR AGT TT-5′) primer pair (Research and Testing, Lubock Texas; [44, 45] For sequencing, forward and reverse sequencing oligonucleotides were designed to contain a unique 8 nt barcode (N), a primer pad (underlined), a linker sequence (italicized), and the Illumina adaptor sequences (bold).

Forward primer: **AATGATACGGCGACCACCGAGATCTACAC-**NNNNNNNN- TATGGTAATT-*GT-*GTGYCAGCMGCCGCGGTAA;

Reverse primer: **CAAGCAGAAGACGGCATACGAGAT-**NNNNNNNN-AGTCAGTCAG- *GG-*CCGYCAATTYMTTTRAGTTT.

Barcode combinations for each sample are provided in Additional file 1: Table S4. Each PCR reaction contained 1 Unit Kapa2G Robust Hot Start Polymerase (Kapa Biosystems, Boston, MA), 1.5 mM MgCl_2_, 10 pmol of each primer, and 1 μL of DNA. The PCR was performed using the following conditions: 95 °C for 2 min, followed by 30 cycles at 95 °C for 10 s, 55 °C for 15 s, 72 °C for 15 s and a final extension step at 72 °C for 3 min. Amplicons were quantified using a Qubit instrument with the Qubit High Sensitivity DNA kit (Invitrogen, Carlsbad, CA). Individual amplicon libraries were pooled, cleaned with Ampure XP beads (Beckman Coulter, Brea, CA), and sequenced using a 300 bp paired-end method on an Illumina MiSeq at RTL Genomics in Lubbock Texas. Raw sequence reads were submitted to NCBI’s Sequence Read Archive under the SRA ID: SRP152555.

### Sequence analysis

Sequencing resulted in a total of 1,251,439 raw reads, which were analyzed using mothur v1.39.5 [46] using the MiSeq SOP accessed on 3/10/2018 [47]. Using the *make.contigs* command, raw sequences were combined into contigs, which were filtered using *screen.seqs* to remove sequences that were > 420 bp or contained ambiguous base calls to reduce PCR and sequencing error. Duplicate sequences were merged with *unique.seqs*, and the resulting unique sequences were aligned to the V4-V5 region of the SILVA SEED alignment reference v123 [48] using *align.seqs*. Sequences were removed if they contained homopolymers longer than 8 bp or did not align to the correct region in the SILVA SEED alignment reference using *screen.seqs*. To further denoise the data, sequences were pre-clustered within each sample allowing a maximum of 3 base pair differences between sequences using *pre.cluster.* Finally, chimeric sequences were removed using VSEARCH [49].

Quality filtered sequences were grouped into OTUs based on 97% sequence identity and classified using the Bayesian classifier and the Greengenes database (August 2013 release of gg_13_8_99) [50] with *classify.seqs*. Sequences that classified as mitochondria, chloroplasts, eukaryotic, or of unknown origin were removed using *remove.lineage.* Samples were rarefied to 6467 sequences per sample, the smallest number of sequences across all collected samples. Singleton abundances were calculated with *filter.shared.* Chao1 diversity [51], Good’s coverage [52], Shannon [53], and inverse Simpson indices were calculated using *summary.single* to quantify coverage and α-diversity.

### α-Diversity

To estimate the microbial diversity within each group, first, rarefaction analyses were performed (Additional file 1: Figure S1) and species richness and diversity indices were calculated (Additional file 1: Table S2.). Variance of the microbial community between and among the different vessels were quantified using a θ_YC_ distance matrix [54].

### β-Diversity

To investigate slow-acting effects of seaweed addition on microbiome communities, we computed Bray-Curtis dissimilarity (β-diversity) [54] between pairs of samples, both within vessels at different time points, and between vessels at identical time points. We also considered Jaccard dissimilarity which only reflects community composition and not relative abundance, but found similar results and so only report the results for Bray-Curtis dissimilarity. We independently computed β-diversity at the genus, family, order, class, and phylum level to assess whether the observed patterns were dependent on taxonomic resolution. For regression statistics, we computed 95% confidence intervals using non-parametric bootstrap resampling, and significance values using permutation tests. Both of the latter approaches gave qualitatively similar results. All analyses were performed using custom-written Java, SQL, and Bash code available at https://github.com/jladau.

### Statistical analysis

Analysis of molecular variance (AMOVA) [55] was used to identify significant differences in community structure between treatment and control vessels using a θ_YC_ distance matrix for the *amova* command in Mothur. The complete results of these statistical tests between each time interval combination is included in the supplementary data.

Gas, VFA, and Euryarchaeota abundance data were analyzed using the linear mixed-effects model (lme) procedure using the R statistical software (version 3.1.1) [56, 57]. The statistical model included treatment, day, time point, treatment×day×time point interactions, treatment×day interactions, treatment×time point interactions, day×time point interactions and the covariate term, with the error term assumed to be normally distributed with mean = 0 and constant variance. Orthogonal contrasts were used to evaluate treatments vs. control, linear, and quadratic effects of treatments. Significant differences among treatments were declared at *p* ≤ 0.05. Differences at 0.05 < *p* ≤ 0.10 were considered as trend towards significance.

## Additional file

Additional file 1: **Table S1.** Quality filtering and OTU distribution at each incubation time. **Table S2.** Diversity indices at each incubation time. **Figures S1A., S1B, S1C** Rarefaction curves of equilibration, control and *A. taxiformis* amended vessels respectively. **Figure S2.** Principle Coordinate Analysis plot. **Table S3.** OTU table. **Table S4.** Raw sequence barcodes for archived 16S rRNA gene amplicon data. **Table S5.** Results of AMOVA and HOMOVA statistical tests. (XLSX 3751 kb)

## Abbreviations

16S rRNA: 16 Svedberg ribosomal ribonucleic acid
AMOVA: Analysis of molecular variance
bp: Base pair
C: Celsius
CH_4_: Methane
Co: Company
CO_2_: Carbon dioxide
DM: Dry matter
DNA: Deoxyribonucleic acid
FID: Flame ionization detector
g: Gram
GC: Gas chromatography
hrs: Hours
IACUC: Institution of Animal Care and Use Committee
ml: Milliliters
OM: Organic matter
OTU: Operational taxonomic unit
PCoA: Principal coordinate analysis
PCR: Polymerase chain reaction
PVC: Poly vinyl chloride
SBR: Super basic ration
SD: Standard deviation
TDN: Total digestible nutrients
TGP: Total gas production
VFA: Volatile fatty acid

## References

[1] Smith PM, Bustamante H, Ahammad H, Clark H, Dong EA, Elsiddig H, Haberl R, Harper J, House M, Jafari O, Masera C, Mbow NH, Ravindranath CW, Rice C, Robledo Abad A, Romanovskaya F, Sperling F, Tubiello F. Agriculture, Forestry and Other Land Use (AFOLU) 2013. In: Climate Change: Mitigation of Climate Change. Contribution of Working Group III to the Fifth Assessment Report of the Intergovernmental Panel on Climate Change. Cambridge, and New York: Cambridge University Press; 2013. https://www.ipcc.ch/site/assets/uploads/2018/02/ipcc_wg3_ar5_chapter11.pdf. Accessed 15 Mar 2018.

[2] Myhre G, Shindell D, Bréon F-M, Collins W, Fuglestvedt J, Huang J, Koch D, Lamarque JF, Lee D, Mendoza B, Nakajima T, Robock A, Stephens G, Takemura T, Zhang H. Anthropogenic and Natural Radiative Forcing 2013. In: Climate Change: The Physical Science Basis. Contribution of Working Group I to the Fifth Assessment Report of the Intergovernmental Panel on Climate. Cambridge and New York: Cambridge University Press; 2013. https://www.ipcc.ch/site/assets/uploads/2018/02/WG1AR5_Chapter08_FINAL.pdf. Accessed 15 Mar 2018.

[3] National Academies of Science Engineering and Medicine (NASEM) (2018). Improving characterization of Anthropogenic methane emissions in the United States.

[4] Henderson C (1980). The influence of extracellular hydrogen on the metabolism of *Bacteroides ruminicola, Anaerovibrio lipolytica* and *Selenomonas ruminantium*. Microbiol.

[5] Czerkawski JW (1986). An introduction to rumen studies.

[6] Beauchemin KA, McGinn SM (2006). Methane emissions from beef cattle: effects of fumaric acid, essential oil, and canola oil. J Anim Sci.

[7] Hristov AN, Oh J, Firkins JL, Dijkstra J, Kebreab E, Waghorn G, Makkar HPS, Adesogan A, Yang W, Lee C, Gerber PJ (2013). Special topics - mitigation of methane and nitrous oxide emissions from animal operations: I. A review of enteric methane mitigation options. J Anim Sci.

[8] Patra A, Park T, Kim M, Yu Z. Rumen methanogens and mitigation of methane emission by anti-methanogenic compounds and substances. J Anim Sci Biotechno. 2017. 10.1186/s40104-017-0145-9. 10.1186/s40104-017-0145-9 PMC5270371 28149512

[9] Gerber PJ, Henderson B, HPS M, Food and Agriculture Organization of the United Nations (2013). Mitigation of greenhouse gas emissions in livestock production: a review of technical options for non-CO_2_ emissions. Rome: food and agriculture organization of the united nations.

[10] Machado L, Magnusson M, Paul NA, Kinley R, de Nys R, Tomkins N (2016). Dose-response effects of *Asparagopsis taxiformis* and *Oedogonium* sp. on *in-vitro* fermentation and methane production. J Appl Phycol.

[11] Nanri A, Mizoue T, Shimazu T, Ishihara J, Takachi R, Noda M, Iso H, Sasazuki S, Sawada N, Tsugane S (2017). Japan public health center-based prospective study group. Dietary patterns and all-cause, cancer, and cardiovascular disease mortality in Japanese men and women: the Japan public health center-based prospective study. PLoS One.

[12] Bansemer MS, Qin JG, Harris JO, Howarth GS, Stone DA (2016). Nutritional requirements and use of macroalgae as ingredients in abalone feed. Rev Aquaculture.

[13] Elizondo-González R, Quiroz-Guzmán E, Escobedo-Fregoso C, Magallón-Servín P, Peña-Rodríguez A (2018). Use of seaweed Ulva lactuca for water bioremediation and as feed additive for white shrimp Litopenaeus vannamei. PeerJ.

[14] Abdul QA, Choi RJ, Jung HA, Choi JS (2016). Health benefit of fucosterol from marine algae: a review. J Sci Food Agr.

[15] Yang YJ, Nam SJ, Kong G, Kim MK (2010). A case–control study on seaweed consumption and the risk of breast cancer. Brit J Nutr..

[16] Corona G, Ji Y, Anegboonlap P, Hotchkiss S, Gill C, Yaqoob P, Spencer JP, Rowland I (2016). Gastrointestinal modifications and bioavailability of brown seaweed phlorotannins and effects on inflammatory markers. Brit J Nutr.

[17] Blunt JW, Copp BR, Munro MH, Northcote PT, Prinsep MR (2013). Marine natural products. Nat Prod Rep.

[18] Machado L, Magnusson M, Paul NA, de Nys R, Tomkins N (2014). Effects of marine and freshwater macroalgae on *In-Vitro* Total gas and methane production. PLoS One.

[19] Hansen H, Hector B, Feldmann J (2003). A qualitative and quantitative evaluation of the seaweed diet of north Ronaldsay sheep. Anim Feed Sci Tech.

[20] Marín A, Casas-Valdez M, Carrillo S, Hernández H, Monroy A, Sanginés L, Pérez-Gil F (2009). The marine algae *Sargassum* spp. (*Sargassaceae*) as feed for sheep in tropical and subtropical regions. Rev Biol Tropic.

[21] Dubois B, Tomkins NW, Kinley RD, Bai M, Seymour S, Paul NA, de Nys R (2013). Effect of tropical algae as additives on rumen *in-vitro* gas production and fermentation characteristics. Am J Plant Sci.

[22] Wang Y, Xu Z, Bach S, McAllister T (2008). Effects of phlorotannins from *Ascophyllum nodosum* (brown seaweed) on *in-vitro* ruminal digestion of mixed forage or barley grain. Anim Feed Sci Tech.

[23] Gonzalez del Val A, Platas G, Basilio A, Cabello A, Gorrochategui J, Suay I, Vicente F, Portillo E, Jimenez del Rio M, Reina GG, Pelaez F (2001). Screening of antimicrobial activities in red, green and brown macroalgae from gran Canaria (Canary Islands, Spain). Int Microbiol.

[24] Yuan YV, Walsh NA (2006). Antioxidant and antiproliferative activities of extracts from a variety of edible seaweeds. Food Chem Toxicol.

[25] Chandini SK, Ganesan P, Bhaskar N (2008). *In-vitro* antioxidant activities of three selected brown seaweeds of India. Food Chem.

[26] Kang JY, Khan MNA, Park NH, Cho JY, Lee MC, Fujii H, Hong YK (2008). Antipyretic, analgesic, and anti-inflammatory activities of the seaweed *Sargassum fulvellum* and *Sargassum thunbergii* in mice. J Ethnopharmacol.

[27] Machado L, Magnusson M, Paul NA, Kinley R, de Nys R, Tomkins N (2016). Identification of bioactives from the red seaweed *Asparagopsis taxiformis* that promote antimethanogenic activity *in-vitro*. J Appl Phycol.

[28] Wood J, Kennedy FS, Wolfe R (1968). Reaction of multihalogenated hydrocarbons with free and bound reduced vitamin B12. Biochemist.

[29] Allen KD, Wegener G, White RH (2014). Discovery of multiple modified F430 coenzymes in methanogens and anaerobic methanotrophic archaea suggests possible new roles for F430 in nature. Appl Environl Microb.

[30] Machado L, Tomkins N, Magnusson M, Midgley D, Rocky dN, Rosewarne C (2018). In vitro response of rumen microbiota to the antimethanogenic red macroalga Asparagopsis taxiformis. Microb Ecol.

[31] Li X, Norman HC, Kinley RD, Laurence M, Wilmot M, Bender H, de Nys R, Tomkins N (2016). *Asparagopsis taxiformis* decreases enteric methane production from sheep. Anim Prod Sci.

[32] Cabeza-Luna I, Carro MD, Fernández-Yepes J, Molina-Alcaide E (2018). Effects of modifications to retain protozoa in continuous-culture fermenters on ruminal fermentation, microbial populations, and microbial biomass assessed by two different methods. Anim Feed Sci Tech..

[33] Holmes DE, Giloteaux L, Orellana R, Williams KH, Robbins MJ, Lovley DR (2014). Methane production from protozoan endosymbionts following stimulation of microbial metabolism within subsurface sediments. Front Microbiol.

[34] Belanche A, de la Fuente G, Newbold CJ (2014). Study of methanogen communities associated with different rumen protozoal populations. FEMS Microb Ecol.

[35] Newbold CJ, Lassalas B, Jouany JP (1995). The importance of methanogens associated with ciliate protozoa in ruminal methane production in vitro. Lett Appl Microbiol.

[36] Morgavi DP, Forano E, Martin C, Newbold CJ (2010). Microbial ecosystem and methanogenesis in ruminants. Animal.

[37] Wolin MJ, Miller TL, Stewart CS (1997). Microbe-microbe interactions. The rumen microbial ecosystem.

[38] Janssen PH (2010). Influence of hydrogen on rumen methane formation and fermentation balances through microbial growth kinetics and fermentation thermodynamics. Anim Feed Sci Tech..

[39] Seymour WM, Campbell DR, Johnson ZB (2005). Relationships between rumen volatile fatty acid concentrations and milk production in dairy cows: a literature study. Anim Feed Sci Tech..

[40] Zaneveld JR, McMinds R, Thurber RV (2017). Stress and stability: applying the Anna Karenina principle to animal microbiomes. Nat Microbiol.

[41] Rey-Crespo F, López-Alonso M, Miranda M (2014). The use of seaweed from the Galician coast as a mineral supplement in organic dairy cattle. Animal.

[42] Czerkawski JW, Breckenridge G (1977). Design and development of a long-term rumen simulation technique (Rusitec). Brit J Nutr..

[43] Oeztuerk H, Schroeder B, Beyerbach M, Breves G (2005). Influence of living and autoclaved yeasts of *Saccharomyces boulardii* on *in-vitro* ruminal microbial metabolism. J Dairy Sci.

[44] Walters W, Hyde ER, Berg-Lyons D, Ackermann G, Humphrey G, Parada A, Gilbert JA, Jansson JK, Caporaso JG, Fuhrman JA, Apprill A (2016). Improved bacterial 16S rRNA gene (V4 and V4-5) and fungal internal transcribed spacer marker gene primers for microbial community surveys. Msystems.

[45] Caporaso JG, Lauber CL, Walters WA, Berg-Lyons D, Huntley J, Fierer N, Owens SM, Betley J, Fraser L, Bauer M (2012). Ultra-high-throughput microbial community analysis on the Illumina HiSeq and MiSeq platforms. ISME J.

[46] Schloss PD, Westcott SL, Ryabin T, Hall JR, Hartmann M, Hollister EB, Lesniewski RA, Oakley BB, Parks DH, Robinson CJ (2009). Introducing mothur: open-source, platform-independent, community-supported software for describing and comparing microbial communities. Appl Environ Microb..

[47] Kozich JJ, Westcott SL, Baxter NT, Highlander SK, Schloss PD (2013). Development of a dual-index sequencing strategy and curation pipeline for analyzing amplicon sequence data on the MiSeq Illumina sequencing platform. Appl Environ Microb..

[48] Quast C, Pruesse E, Yilmaz P, Gerken J, Schweer T, Yarza P, Peplies J, Glockner FO (2013). The SILVA ribosomal RNA gene database project: improved data processing and web-based tools. Nuc Acids Res.

[49] Edgar RC, Haas BJ, Clemente JC, Quince C, Knight R (2011). UCHIME improves sensitivity and speed of chimera detection. Bioinformatics.

[50] DeSantis TZ, Hugenholtz P, Larsen N, Rojas M, Brodie EL, Keller K, Huber T, Dalevi D, Hu P, Andersen GL (2006). Greengenes, a chimera-checked 16S rRNA gene database and workbench compatible with ARB. Appl Environ Microb.

[51] Chao A (1984). Nonparametric estimation of the number of classes in a population. Scan J Stat.

[52] Good IJ (1953). The population frequencies of species and the estimation of population parameters. Biometrika.

[53] Shannon CE (1948). A mathematical theory of communication. Bell Sys Tech J.

[54] Yue JC, Clayton MK (2005). A similarity measure based on species proportions. Comm Stat-theory Meth.

[55] Bray JR, Curtis JT (1957). An ordination of the upland forest communities of southern Wisconsin. Ecol Monogr.

[56] Excoffier L, Smouse PE, Quattro JM (1992). Analysis of molecular variance inferred from metric distances among DNA haplotypes: application to human mitochondrial DNA restriction data. Genetics.

[57] Team RC (2014). R: a language and environment for statistical computing.

