# Correction to: Effect of the macroalgae *Asparagopsis taxiformis* on methane production and rumen microbiome assemblage

**DOI:** 10.1186/s42523-019-0005-3

**Published:** 2019-03-27

**Authors:** Breanna Michell Roque, Charles Garrett Brooke, Joshua Ladau, Tamsen Polley, Lyndsey Jean Marsh, Negeen Najafi, Pramod Pandey, Latika Singh, Robert Kinley, Joan King Salwen, Emiley Eloe-Fadrosh, Ermias Kebreab, Matthias Hess

**Affiliations:** 1 Department of Animal Science, University of California, 2251 Meyer Hall, Davis, CA 95616 USA; 2 Department of Energy Joint Genome Institute, 2800 Mitchell Drive, Walnut Creek, CA 94598 USA; 3 Department of Population Health and Reproduction, School of Veterinary Medicine, One Shields Avenue, Davis, CA 95616 USA; 4 Department of Earth System Science, Stanford University, 450 Serra Mall, Stanford, CA 94305 USA; 5 Agriculture and Food, Commonwealth Scientific and Industrial Research Organisation (CSIRO), Building 145 James Cook Drive, James Cook University, Townsville, QLD 4811 Australia

**Correction to: Animal Microbiome (2019) 1:3**

**https://doi.org/10.1186/s42523-019-0004-4**

Robert Kinley was not listed among the authors in the original article [1]. The correct author list is as follows:

Breanna Michell Roque, Charles Garrett Brooke, Joshua Ladau, Tamsen Polley, Lyndsey Jean Marsh, Negeen Najafi, Pramod Pandey, Latika Singh, Robert Kinley, Joan King Salwen, Emiley Eloe-Fadrosh, Ermias Kebreab and Matthias Hess*.

The original article has been corrected.
